# Real-world comparative effectiveness of triplets containing bortezomib (B), carfilzomib (C), daratumumab (D), or ixazomib (I) in relapsed/refractory multiple myeloma (RRMM) in the US

**DOI:** 10.1007/s00277-021-04534-8

**Published:** 2021-05-10

**Authors:** Faith Davies, Robert Rifkin, Caitlin Costello, Gareth Morgan, Saad Usmani, Rafat Abonour, Antonio Palumbo, Dorothy Romanus, Roman Hajek, Evangelos Terpos, Dasha Cherepanov, Dawn Marie Stull, Hui Huang, Xavier Leleu, Jesus Berdeja, Hans C. Lee, Katja Weisel, Michael Thompson, Mario Boccadoro, Jeffrey Zonder, Gordon Cook, Noemi Puig, Jorge Vela-Ojeda, Eileen Farrelly, Aditya Raju, Marlo Blazer, Ajai Chari

**Affiliations:** 1 NYU Langone Health, New York, NY USA; 2 Rocky Mountain Cancer Centers, Denver, CO USA; 3 Moores Cancer Center, University of California, San Diego, CA USA; 4 Carolinas Healthcare System, Charlotte, NC USA; 5 Indiana University, Indianapolis, IN USA; 6 Millennium Pharmaceuticals, Inc., a wholly owned subsidiary of Takeda Pharmaceutical Company Limited, Cambridge, MA USA; 7 University Hospital Ostrava and Faculty of Medicine, University of Ostrava, Ostrava, Czech Republic; 8 University of Athens School of Medicine, Athens, Greece; 9 CHU la Miletrie, Poiters, France; 10 Tennessee Oncology, Sarah Cannon Research Institute, Nashville, TN USA; 11 University of Texas, MD Anderson Cancer Center, Houston, TX USA; 12 Department of Oncology, Hematology and Bone Marrow Transplantation with Section of Pneumology, University Medical Center Hamburg-Eppendorf, Hamburg, Germany; 13 Aurora Cancer Center, Advocate Aurora Health, Milwaukee, WI USA; 14 Azienda Ospedaliera Citta della Salute e della Scienza, Torino, Italy; 15 Karmanos Cancer Institute, Detroit, MI USA; 16 Leeds Teaching Hospitals NHS Trust, Leeds, UK; 17 Salamanca University Hospital, Salamanca, Spain; 18 Hospital de Especialidades Centro Medico Nacional la Raza, Mexico City, Mexico; 19 Xcenda, Palm Harbor, FL USA; 20 Icahn School of Medicine at Mount Sinai, New York, NY USA

**Keywords:** Relapsed refractory multiple myeloma, Proteasome inhibitor triplet therapy, Real-world, Bortezomib, Carfilzomib, Ixazomib, Daratumumab, Lenalidomide, Pomalidomide

## Abstract

Multiple available combinations of proteasome inhibitors, immunomodulators (IMIDs), and monoclonal antibodies are shifting the relapsed/refractory multiple myeloma (RRMM) treatment landscape. Lack of head-to-head trials of triplet regimens highlights the need for real-world (RW) evidence. We conducted an RW comparative effectiveness analysis of bortezomib (V), carfilzomib (K), ixazomib (I), and daratumumab (D) combined with either lenalidomide or pomalidomide plus dexamethasone (Rd or Pd) in RRMM. A retrospective cohort of patients initiating triplet regimens in line of therapy (LOT) ≥ 2 on/after 1/1/2014 was followed between 1/2007 and 3/2018 in Optum’s deidentified US electronic health records database. Time to next treatment (TTNT) was estimated using Kaplan-Meier methods; regimens were compared using covariate-adjusted Cox proportional hazard models. Seven hundred forty-one patients (820 patient LOTs) with an Rd backbone (VRd, *n* = 349; KRd, *n* = 218; DRd, *n* = 99; IRd, *n* = 154) and 348 patients (392 patient LOTs) with a Pd backbone (VPd, *n* = 52; KPd, *n* = 146; DPd, *n* = 149; IPd, *n* = 45) in LOTs ≥2 were identified. More patients ≥75 years received IRd (39.6%), IPd (37.8%), and VRd (36.7%) than other triplets. More patients receiving VRd/VPd were in LOT2 vs other triplets. Unadjusted median TTNT in LOT ≥ 2: VRd, 13.9; KRd, 8.7; IRd, 11.4; DRd, not estimable (NE); and VPd, 12.0; KPd, 6.7; IPd, 9.5 months; DPd, NE. In covariate-adjusted analysis, only KRd vs DRd was associated with a significantly higher risk of next LOT initiation/death (HR 1.72; *P* = 0.0142); no Pd triplet was significantly different vs DPd in LOT ≥ 2. Our data highlight important efficacy/effectiveness gaps between results observed in phase 3 clinical trials and those realized in the RW.

**Supplementary Information:**

The online version contains supplementary material available at 10.1007/s00277-021-04534-8.

## Introduction

Multiple myeloma (MM) is the second most common hematologic malignancy, with an estimated incidence of 32,000 cases and 13,000 deaths in 2019 in the United States (US) [1]. Recent treatment advances in MM have led to an increased overall survival rate; however, the majority of MM patients require subsequent treatment for relapsed and/or refractory multiple myeloma (RRMM) [2, 3].

While traditionally, triplet regimens containing a bortezomib-based backbone (i.e., combined with dexamethasone and either lenalidomide [VRd] or cyclophosphamide [VCd]) have been the mainstay of therapy in RRMM [3], the addition of various proteasome inhibitors (PIs), immunomodulators (IMIDs), and monoclonal antibodies (mABs) in multiple myeloma (MM) is rapidly shifting the RRMM treatment landscape. Triplets containing ixazomib, carfilzomib, or daratumumab in combination with the lenalidomide and dexamethasone (Rd) backbone have demonstrated superior clinical outcomes compared to the doublet therapy of Rd in randomized clinical trials (RCTs) [4–7]. This RCT-based evidence of improved outcomes has led to the increased use of triplet therapy with a PI or mAB combined with Rd in routine clinical practice for patients with RRMM [8]. Furthermore, with lenalidomide use moving to earlier lines of therapy, this has also contributed to the increasing use of subsequent pomalidomide-dexamethasone (Pd) triplet therapy [8, 9].

Despite plentiful RCT evidence on specific regimens, lack of head-to-head trials of these agents renders treatment choices for RRMM patients difficult. Cross-trial comparisons are typically not appropriate for several reasons, including differences in study designs and populations across trials. Differences in eligibility criteria across trials can result in imbalances between trial populations, introducing variations in outcomes that are not treatment related [10]. For example, the same control arm regimen from the recent hallmark trials of the aforementioned triplet combinations in RRMM yielded different median progression-free survival (PFS), with values ranging from 14.5 to 17.6 months for Rd [4–7]. Differences in trial population were further demonstrated by a substantial range in proportions of real-world RRMM patients (45 to 75%) not meeting eligibility criteria for inclusion in these pivotal trials [11]. Similarly, the PFS for Pd backbone regimens can range from 4.7 to 7.1 months [12–14].

Importantly, the underrepresentation of older adults and patients with prevalent comorbidities in trials highlights another concern regarding the generalizability of results from RCTs in MM to real-world patients. Translating trial-based efficacy results to patients treated in routine care whose characteristics are not represented in these trials may result in notable gaps in realized real-world effectiveness [11, 15]. To help inform treatment decision-making in routine care, we aimed to conduct a real-world comparative effectiveness analysis of triplet regimens containing bortezomib (V), carfilzomib (K), ixazomib (I), or daratumumab (D) in patients with RRMM, focusing on triplet regimens with an IMiD and dexamethasone (Rd or a Pd) backbone.

## Methods

### Data source

This was a retrospective cohort study using Optum’s deidentified electronic health record (EHR) database from 1/1/2007 to 3/31/2018. This is a general population-representative dataset with data from all 50 states in the US and over 140,000 providers, 6500 clinics, and 600 hospitals [16]. Data are certified as deidentified in line with the US Health Insurance Portability and Accountability Act statistical deidentification rules. This study was approved by the Advarra Institutional Review Board.

### Study design and population

Included patients were adults diagnosed with MM and treated with at least 1 prior line of therapy (LOT) initiating a triplet regimen containing V, K, I, or D combined with either an Rd or Pd backbone on or after 1/1/2014. The first diagnosis for MM (International Classification of Diseases, 9th Revision [ICD-9] code: 203.0x; 10th Revision [ICD-10] code: C90.0x) between 1/1/2007 and 3/31/2018 was designated as the diagnosis date with a 6-month period prior to the first diagnosis window where no MM diagnoses were recorded. Triplet regimens were first categorized into V-, K-, I-, or D-based; regimen combinations that included D plus a PI (i.e., V, K, or I) were categorized as D-based triplet regimens. Next, among these, Rd or Pd backbone triplet regimens were selected. To holistically examine the comparative effectiveness of the index regimens as reflected by their real-world use, irrespective of sequencing, we opted to include recurrent use of the index regimens within a patient for this analysis (e.g., VRd in LOT 2 followed by IRd in LOT 3) rather than restricting to only the first use of the index regimen (e.g., VRd in LOT 2 in the previous example). Hence, the unit of measure was patient LOT, and the index date for each triplet LOT of interest was the first date that each triplet regimen was initiated in LOT 2 or later (LOT ≥ 2). The 6-month period prior to each index date, termed the baseline period, was used to characterize the study patients.

Only patients with continuous care in an integrated delivery network for 6 months prior to the MM diagnosis date through at least initiation of index LOT were included to ensure data completeness. Patients with receipt of a stem cell transplant (SCT) during the index LOT and evidence of anticancer therapy or SCT during the 6-month period prior to the first MM diagnosis date were excluded to minimize the possibility of including prevalent cases with incomplete histories.

**Fig. 1:**
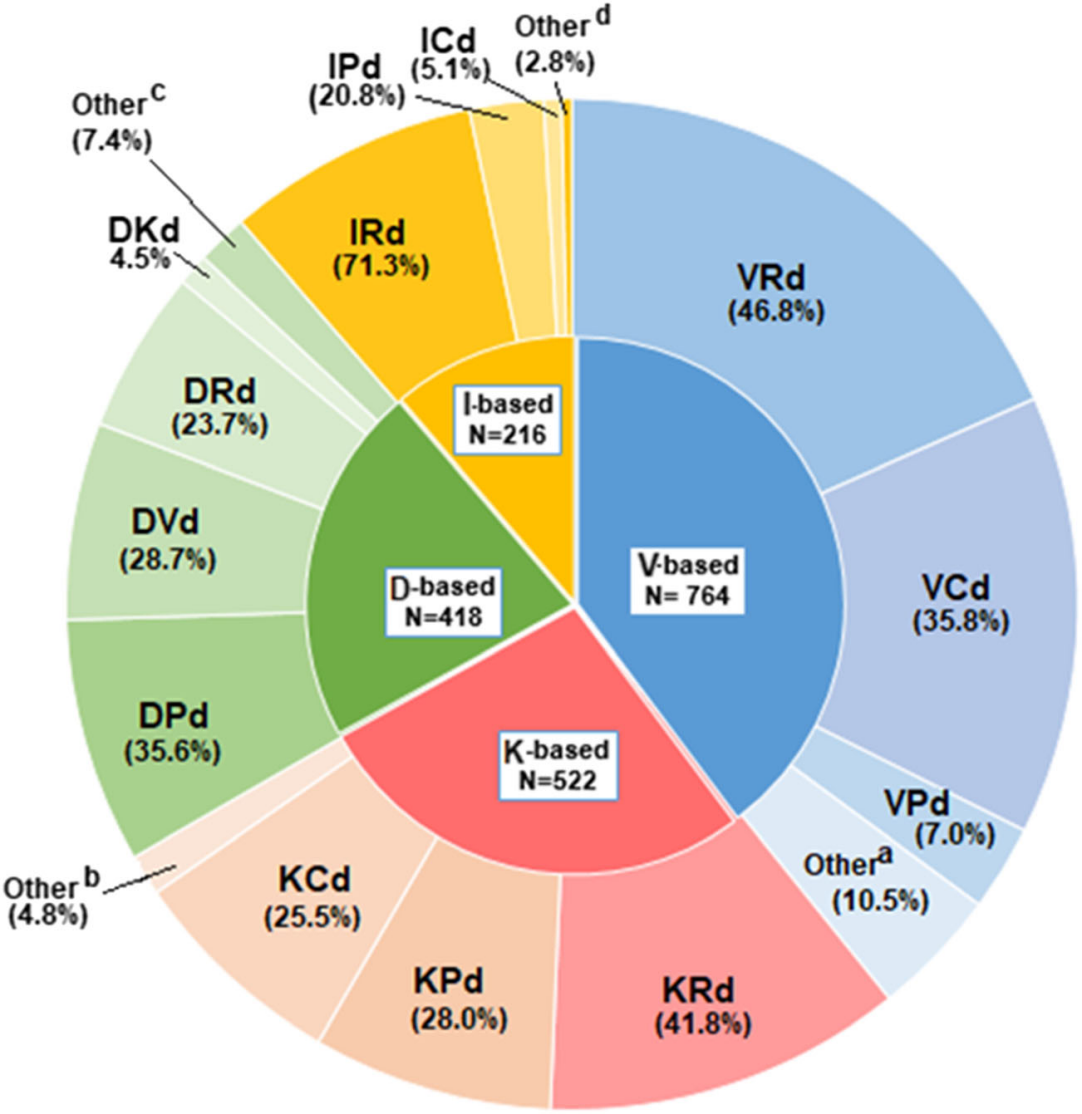
Regimen composition for LOTs ≥ 2. ^a^Other includes bendamustine, doxorubicin, etoposide, melphalan, panobinostat, thalidomide, and vincristine. ^b^Other includes doxorubicin, melphalan, panobinostat, and thalidomide. ^c^Other includes cyclophosphamide, ixazomib, melphalan, thalidomide, and panobinostat. ^d^Other includes bendamustine, melphalan, and thalidomide. Each component of “other” contributed < 3.5% to the total within each group. Key: C, cyclophosphamide; d, dexamethasone; D, daratumumab; I, ixazomib; K, carfilzomib; V, bortezomib; P, pomalidomide; R, lenalidomide

Patients were followed longitudinally until death, loss to follow-up, or end of study period (3/31/2018), whichever occurred first (Fig. 1, Supplemental Appendix)

### Study variables

Baseline demographic and clinical characteristics were assessed longitudinally via diagnosis codes, lab values, and standardized fields within the EHR. Patients with missing data were categorized separately.

The algorithm for LOT determination in the EHR was developed in collaboration with several hematology/oncology specialists to proxy the definition of an LOT within the RCTs and in accordance with the NCCN Guidelines for treatment of MM [9, 17, 18] (see Appendix). Time to index LOT (in months) was defined as the time from the MM diagnosis date to the start of the index triplet regimen. Treatment-free interval (TFI) (in months) was defined as the time from the end of the previous LOT to the start of the next LOT, with a TFI of ≤ 60 days defined as “refractory to last therapy.” Refractory status to any PIs or any IMIDs was defined as follows: duration of therapy (DOT) of the IMID or PI within a regimen was ≤ 60 days and the PI/IMID was not in next LOT, or the TFI between LOTs was ≤ 60 days and the PI or IMID was not in the subsequent LOT [19].

### Outcomes

The primary outcome of interest was time to next therapy (TTNT), which is a surrogate marker for PFS in real-world analyses [20, 21]. TTNT was defined as the time from the start of the index regimen to initiation of subsequent line of therapy or death, whichever occurred earlier. Patients were censored if they did not have an event (start of next LOT/death) by the date of last EHR activity or end of the study period, whichever occurred earlier.

DOT of the index regimen was also evaluated and defined as the time from initiation of the index regimen to discontinuation of the last drug in the regimen plus a run-out period. The run-out date for infused/injected drugs was the latest date of administration + 30 days, and for orally administered drugs, it was the fill date + (days’ supply −1). Patients were censored if they did not initiate a subsequent therapy and had < 180 days of follow-up after the latest date of administration of the index regimen (follow-up was terminated at the date of last EHR activity, death, or end of study period, whichever occurred earlier).

### Statistical analysis

Baseline characteristics of the study population by treatment category were presented as counts and percentages for categorical variables and means, standard deviations (SDs), medians, and interquartile ranges (IQRs) for continuous variables. To adequately account for correlation between observations (due to the patient LOT-level analyses), statistical differences in baseline characteristics were assessed using generalized estimating equation models. For binary variables, a binomial distribution and log-link function were specified; for other categorical variables, a multinomial distribution and cumulative log-link were specified; and for continuous variables, ranks were assigned to the observations and compared across treatment groups using a GENMOD model.

TTNT and DOT were estimated using Kaplan-Meier (KM) methods. The risk of initiation of next line of therapy after start of the index triplet regimen or death for analyses of TTNT and the risk of discontinuation of the index triplet regimen for analyses of DOT were estimated with hazard ratios (HRs) and compared using stratified (by LOT) Cox proportional hazard (PH) models. To account for correlation between observations in the PH models (due to the patient LOT-level analyses), between-clusters variance was computed from robust sandwich estimators for adjusted standard errors in the Cox PH analyses. Use of the standard Cox PH estimation of variance, without robust sandwich estimators, would have increased the chance of type I error and thus the possibility of erroneously claiming a significant finding. All survival analyses were stratified by LOT (2, 3, ≥ 4). In addition, we conducted covariate-adjusted multivariate Cox PH analyses of TTNT. The following variables were included in multivariate models: regimen type (V-, K-, I-, or D-based); age (18–64, 65–74, ≥ 75 years); Charlson comorbidity index (CCI) (0, 1, ≥ 2); Eastern Cooperative Oncology Group (EGOG) performance status (PS) (0–1, 2–4, unknown); presence of CRAB symptoms (hypercalcemia, renal insufficiency, anemia, bone disease); presence of peripheral neuropathy, and prior SCT; cytogenetic risk (standard risk/unknown, high risk); International Staging System (ISS) stage (I/II, III, unknown); prior treatment exposure (PI, IMID, PI+IMID, and none); prior daratumumab exposure; PI- and/or IMID-refractory status (PI-refractory, IMID-refractory, PI- + IMID-refractory, and not refractory); time from diagnosis to start of index line of therapy (months); refractory to prior line of therapy (TFI of ≤ 60 days vs > 60 days); time from start of LOT1 to start of LOT2 (months) as a proxy for time to first relapse; year of diagnosis (2007–2011, 2012–2015, 2016–2017); and setting of care (community, academic, unknown).

The main analysis was based on the cohort of eligible patients receiving one of the V-, K-, I-, or D-based triplet regimens with either an Rd (i.e., VRd, KRd, IRd, or DRd) or a Pd (i.e., VPd, KPd, IPd, or DPd) backbone in LOT ≥ 2. Rd and Pd backbone triplet cohorts were analyzed separately. We also performed an exploratory subgroup analysis for patients who received the regimens of interest in earlier LOTs (i.e., in LOTs 2–3).

## Results

### Regimen composition

Overall, there were 1432 patients with 1902 patient LOTs containing V-, K-, D-, or I-based triplet regimens in LOT ≥ 2 (V-based, *n* = 746; K-based, *n* = 522; D-based, *n* = 418; I-based, *n* = 216). Among all patients receiving one of the triplet regimens of interest in LOT ≥ 2, the compositions of the regimens within each triplet category are shown in Fig. 1. Rd was the predominant backbone for the V-, K-, and I-based triplet regimens: 46.8%, 41.8%, and 71.3%, respectively. Among D-based regimens, Pd (35.6%), Rd (23.7%), and Vd (28.7%) backbones were relatively evenly distributed.

### Baseline characteristics: Rd and Pd backbone triplets

There were 741 patients with 820 patient LOTs in combination with an Rd backbone (VRd, *n* = 349; KRd, *n* = 218; IRd, *n* = 154; DRd, *n* = 99) and 348 patients with 392 patient LOTs in combination with a Pd backbone (DPd, *n* = 149; KPd, *n* = 146; VPd, *n* = 52; IPd, *n* = 45) in LOTs ≥ 2 (Table 1).

**Table 1:** Baseline clinical and treatment characteristics by regimens for LOT ≥ 2

Variable (%)	Rd backbone cohort, *N* = 820					Pd backbone cohort, *N* = 392				
	VRd (*N* = 349)	KRd (*N* = 218)	DRd (*N* = 99)	IRd (*N* = 154)	*P*-value	VPd (*N* = 52)	KPd (*N* = 146)	DPd (*N* = 149)	IPd (*N* = 45)	*P*-value
Age, median years (IQR)^a^	70 (62, 78)	64 (56, 74)	70 (61, 75)	70 (62, 79)	< 0.01	70 (64, 75)	66 (60, 73)	68 (60, 74)	67 (63, 77)	0.07
Age group, years										
18–64	34.1	50.9	37.4	36.4	< 0.01	26.9	43.2	36.9	35.6	0.10
65–74	29.2	27.5	36.4	24.0		44.2	37.7	39.6	26.7	
≥ 75	36.7	21.6	26.3	39.6		28.9	19.2	23.5	37.8	
CCI score^b,c^										
0	28.9	27.0	27.3	39.0	0.06	32.7	23.3	23.5	42.2	0.10
1	10.0	9.2	15.2	11.0		7.7	16.4	9.4	11.1	
≥ 2	61.0	63.8	57.6	50.0		59.6	60.3	67.1	46.7	
ECOG PS										
0–1	15.5	18.4	21.2	15.6	0.30	42.3	18.5	22.1	13.3	0.02
2–4	2.0	3.2	5.1	2.0		0.0	4.1	4.0	2.2	
Unknown	82.5	78.4	73.7	82.5		57.7	77.4	73.8	84.4	
Cytogenetics^d^										
High risk	11.8	25.7	10.1	14.9	< 0.01	17.3	26.0	33.6	11.1	< 0.01
Not high risk/unk	88.3	74.3	89.9	85.1		82.7	74.0	66.4	88.9	
CRAB symptoms^b,e^										
Any	78.5	84.4	78.8	63.6	< 0.01	76.9	86.3	86.6	62.2	0.01
Hypercalcemia	10.6	12.8	10.1	6.5	0.22	9.6	14.4	15.4	4.4	0.06
RI	47.9	42.7	52.5	29.2	< 0.01	42.3	44.5	53.0	20.0	< 0.01
Anemia	65.6	75.2	64.7	56.5	< 0.01	69.2	80.1	82.6	46.7	< 0.01
Bone disease	19.8	25.7	26.3	18.2	0.18	26.9	27.4	29.5	17.8	0.40
Baseline PN	11.5	22.5	31.3	19.5	< 0.01	9.6	24.0	22.2	31.5	< 0.01
Baseline CVD	28.6	26.6	25.3	19.5	0.14	26.9	23.3	31.5	13.3	0.05
ISS stage										
I/II	20.1	20.6	16.2	11.0	0.64	15.4	27.4	18.8	15.6	NE
III	5.4	6.0	2.0	2.6		7.7	6.2	6.0	0.0	
Unk	74.5	73.4	81.8	86.4		76.9	66.4	75.2	84.4	
Site of care										
Community	45.0	39.5	37.4	44.8	0.11	32.7	28.1	36.9	37.8	0.09
Academic	21.2	18.4	16.2	18.2		25.0	31.5	16.8	28.9	
Unknown	33.8	42.2	46.5	37.0		42.3	40.4	46.3	33.3	
Year of diagnosis										
2007–2011	18.1	18.8	13.1	20.1	0.48	42.3	32.2	24.2	33.3	0.06
2012–2015	63.3	63.3	64.6	61.7		51.9	60.3	65.8	60.0	
2016–2017	18.6	17.9	22.2	18.2		5.8	7.5	10.1	6.7	
Index LOT										
2	69.3	46.3	30.3	40.9	< 0.01	28.6	20.6	11.4	17.8	< 0.01
3	19.8	25.2	25.3	29.2		25.0	24.0	16.1	26.7	
4	7.5	16.1	18.2	16.9		26.9	24.7	28.2	15.6	
≥ 5	3.4	12.4	26.3	13.0		19.2	30.8	44.3	40.0	
Prior exposure to IMIDs or PIs										
IMID and PI	31.5	67.0	67.7	57.8	< 0.01	71.2	89.7	86.6	75.6	0.02
PI only	42.1	26.2	21.2	13.0	< 0.01	86.5	93.2	96.0	97.8	0.13
IMID only	23.5	5.5	10.1	27.9	< 0.01	11.5	2.7	8.7	22.2	< 0.01
Prior dara	0.0	4.6	24.2	5.8	NE	1.9	9.6	28.9	2.2	< 0.01
Refractory status to PIs and/or IMIDs										
Both IMID and PI	3.4	21.6	31.3	22.1	< 0.01	25.0	59.6	68.5	44.4	0.29
IMID only	8.0	6.4	3.0	9.7		38.5	15.1	11.4	28.9	
PI only	3.4	53.2	39.4	25.3		7.7	10.3	14.8	13.3	
Neither	85.1	18.8	26.3	42.9		28.9	15.1	5.4	13.3	
Refractory to prior LOT^f^	80.0	85.3	81.8	70.8	0.01	78.9	84.9	87.3	86.7	0.60
Prior SCT	15.8	33.5	41.4	16.9	< 0.01	32.7	31.5	38.9	28.9	0.67
Time from diagnosis to index LOT										
Median (IQR)	17.9 (8.3, 35.9)	21.0 (10.6, 42.1)	35.7 (18.8, 52.3)	32.7 (16.5, 55.2)	< 0.01	32.9 (24.5, 59.5)	32.1 (15.9, 55.2)	38.0 (24.8, 60.6)	52.8 (31.6, 67.2)	0.02
Time from start of LOT1 to start of LOT2										
Median (IQR)	10.1 (5.3, 17.9)	9.9 (5.3, 16.6)	12.6 (6.9, 25.5)	15.0 (9.3, 24.0)	< 0.01	14.0 (7.7, 25.5)	11.3 (6.5, 20.7)	12.6 (7.1, 23.3)	17.3 (7.9, 24.7)	0.21

^a^Age at the time of initiation of LOT ≥ 2

^b^Baseline presence is relative to 6 months prior to initiation of index LOT with the value closest to index LOT initiation, recorded

^c^Source: Quan 2002 [22]

^d^High-risk cytogenetics were defined as presence of del[17p], t[4;14], t[14;16], and/or 1q21 gain

^e^CRAB definitions: hypercalcemia (identified via ICD-9/10 code or lab value indicating corrected serum calcium > 11 mg/dL), renal insufficiency (defined as lab value indicating serum creatinine > 2 mg/dL or creatinine clearance < 40 mL/min), anemia (identified via ICD-9/10 code or hemoglobin level < 10 gm/dL), and bone lesions (proxied by fracture, radiation, bone-directed surgery, or spinal cord compression) (Nash 2016) [23]

^f^Refractory status to prior LOT was defined as a TFI of < 60 days between previous LOT and index LOT

Key: *CCI*, Charlson comorbidity index; *Dara*, daratumumab; *DPd*, daratumumab, pomalidomide, dexamethasone; *DRd*, daratumumab, lenalidomide, dexamethasone; *IMID*, immunomodulatory drug; *IPd*, ixazomib, pomalidomide, dexamethasone; *IQR*, interquartile range; *IRd*, ixazomib, lenalidomide, dexamethasone; *ISS*, International Staging System; *KPd*, carfilzomib, pomalidomide, dexamethasone; *KRd*, carfilzomib, lenalidomide, dexamethasone; *LOT*, line of therapy; *PI*, proteasome inhibitor; *RI*, renal insufficiency; *SCT*, stem cell transplant; *sxs*, symptoms; *TFI*, treatment-free interval; *Unk*, unknown; *VPd*, bortezomib, pomalidomide, dexamethasone; *VRd*, bortezomib, lenalidomide, dexamethasone

Within both of these IMID-backbone cohorts, there was a statistically significant difference in age between groups. A larger proportion of patients selected for KRd were < 65 years of age (50.9%) than in the other Rd triplet groups (DRd, 37.4%; IRd, 36.4%; VRd, 34.1%). Similar differences in age distributions were observed for Pd backbone regimens, although the difference was not significant. Among all triplet regimens, the greatest proportions of patients ≥ 75 years of age appeared among those receiving IRd (39.6%), IPd (37.8%), and VRd (36.7%) (Table 1).

Within the Rd backbone cohort, fewer patients receiving IRd had any CRAB symptoms at the start of index LOT (KRd, 84.4%; DRd, 78.8%; VRd, 78.5%; IRd, 63.6%; *P<*0.01). Additionally, high-risk cytogenetic disease was more common in the KRd group (25.7%) than in the other groups (IRd, 14.9%; VRd, 11.8%; DRd, 10.1%; *P <* 0.01). Fewer patients treated with VRd (31.5%) had prior exposure to both an IMID and a PI prior to initiating therapy than those with DRd (67.7%), KRd (67.0%), or IRd (57.8%; *P* < 0.01). Finally, fewer VRd patients were refractory to both an IMID and a PI (DRd, 31.3%; IRd, 22.1%; KRd, 21.6%; VRd, 3.4%; *P <* 0.01).

Correspondingly, within the Pd backbone cohort, the IPd group had a lower proportion of patients with CRAB symptoms: 62.2% vs DPd, 86.6%; KPd, 86.3%; VPd, 76.9%; *P* = 0.01). However, high-risk cytogenetics were more common in both the KPd (26.0%) and the DPd (33.6%) groups than in the VPd (17.3%) or IPd (11.1%) groups (*P* < 0.01) (Table 1).

More patients receiving VRd and VPd received these triplets as second-line therapy vs the other Rd and Pd backbone groups, as noted in Table 1. Within the Rd backbone cohort, the time from diagnosis to initiation of the index triplet regimen was also shortest for those patients receiving VRd (median, 17.9 months) vs those receiving other triplet regimens (KRd, 21.0 months; DRd, 35.7 months; IRd, 32.7 months; *P* < 0.01).

These differences between regimens in time from diagnosis to initiation of index triplet LOT regimen, prior IMID/PI exposure, and PI/IMiD refractory status were consistent for the Pd backbone cohort. Notably, almost 24.2% of patients receiving DRd and 28.9% of patients receiving DPd had prior exposure to daratumumab.

The median follow-up for all patients with Rd or Pd backbone triplets was 13.0 and 9.5 months, respectively. This varied by treatment group: for the Rd backbone triplets, median follow-up was 16.8 months in the VRd group, 13.6 months in the KRd group, 10.4 months in the IRd group, and 9.2 months in the DRd group. For the Pd backbone triplets, median follow-up was 12.5 months in the KPd group, 12.1 months in the VPd group, 10.2 months in the IPd group, and 7.2 months in the DPd group.

### Unadjusted duration of therapy

Among patients in the Rd cohort receiving the regimens of interest in LOT ≥ 2, the VRd group had the longest DOT (10.7 months), followed by IRd (8.5 months) and KRd (7.0 months) (*P* = 0.0034). The median DOT for those treated with DRd was not estimable with the current follow-up. Among patients receiving a Pd backbone, again, VPd had the longest median DOT of 9.7 months followed by IPd (8.8 months), KPd (6.7 months), and DPd (6.0 months), but the difference was not significant (*P* = 0.4784).

### Unadjusted time to next therapy

In this real-world analysis, median TTNT in LOT ≥ 2 was 13.9 months for VRd, 11.4 months for IRd, and 8.7 months for KRd. With the current follow-up in the DRd group, the median TTNT was not estimable for this regimen. In further exploration of patients who received an Rd backbone triplet in earlier lines (LOT 2 or 3), the median TTNT was 16.6 months for IRd (*n* = 108; median follow-up was 11.1 months), 14.1 months for VRd (*n* = 311; median follow-up was 16.6 months), and 8.8 months for KRd (*n* = 156; median follow-up was 14.1 months) (*P* = 0.0040) (based on the log-rank test). The median TTNT in LOTs 2 to 3 was not estimable for DRd (*n* = 55), owing to a shorter follow-up time (median follow-up 8.2 months).

**Fig. 2:**
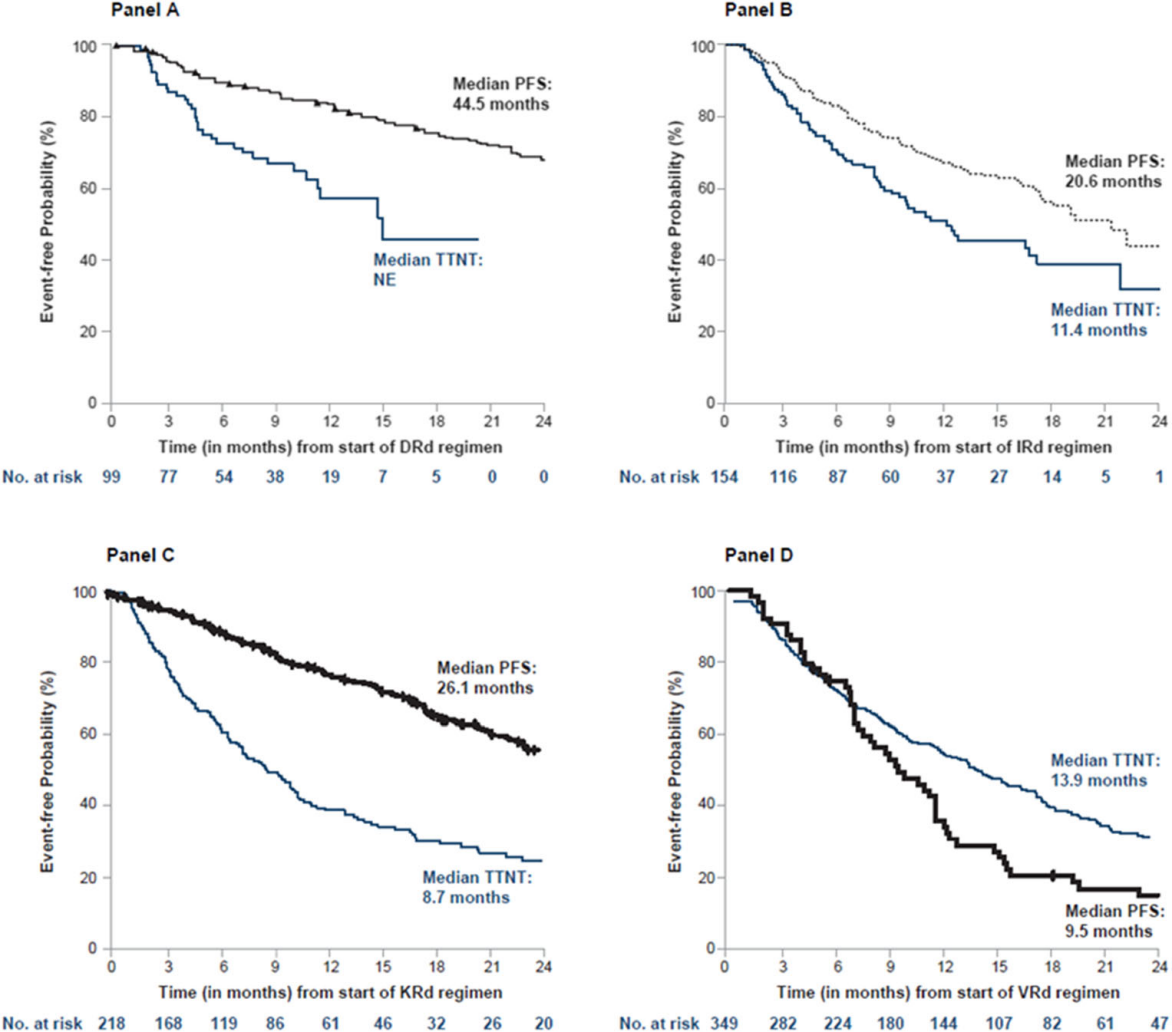
Real-world unadjusted TTNT vs PFS from hallmark trials in LOT ≥ 2 for DRd (**a**), IRd (**b**), KRd (**c**), and VRd (**d**). ^a^An event in the embedded clinical trials was defined as progression or death; an event in the real-world population in this study was defined as the start of the next line of therapy or death (as a proxy for PFS). ^b^Embedded PFS KM curves were adapted from **a** Bahlis NJ, et al. *Leukemia*. 2020 Jan 30. doi: 10.1038/s41375-020-0711-6; **b** Moreau P, et al. *N Engl J Med*. 2016;374:1621-1634; **c** Siegel DS, et al. *J Clin Oncol*. 2018 Mar 10;36(8):728-734; **d** Richardson PG, et al. *Blood*. 2014 Mar 6;123(10):1461-9. Key: DRd, daratumumab, lenalidomide, dexamethasone; IRd, ixazomib, lenalidomide, dexamethasone; KM, Kaplan-Meier; KRd, carfilzomib, lenalidomide, dexamethasone; LOT, line of therapy; PFS, progression-free survival; Rd, lenalidomide, dexamethasone; TTNT, time to next therapy; VRd, bortezomib, lenalidomide, dexamethasone

Comparing these results to PFS reported from clinical trials, this analysis indicated that patients who were selected to receive one of the Rd backbone triplets in the real world experienced notably shorter TTNT compared to the PFS reported among clinical trial patients, except for VRd (Fig. 2) [5, 24–26].

The median unadjusted TTNT for those in the Pd backbone cohort receiving treatment in LOT ≥2 was 12.0 months for VPd, 9.5 months for IPd, 6.7 months for KPd, and NE for DPd (*P* = 0.0946, based on the log-rank test). TTNT for patients who received a Pd backbone in earlier LOTs (LOT 2-3) could not be evaluated due to small sample sizes across cohorts (DPd, *n* = 41; IPd, *n* = 20; KPd, *n* = 65; VPd, *n* = 28).

### Adjusted time to next therapy: multivariate analysis

**Fig. 3:**
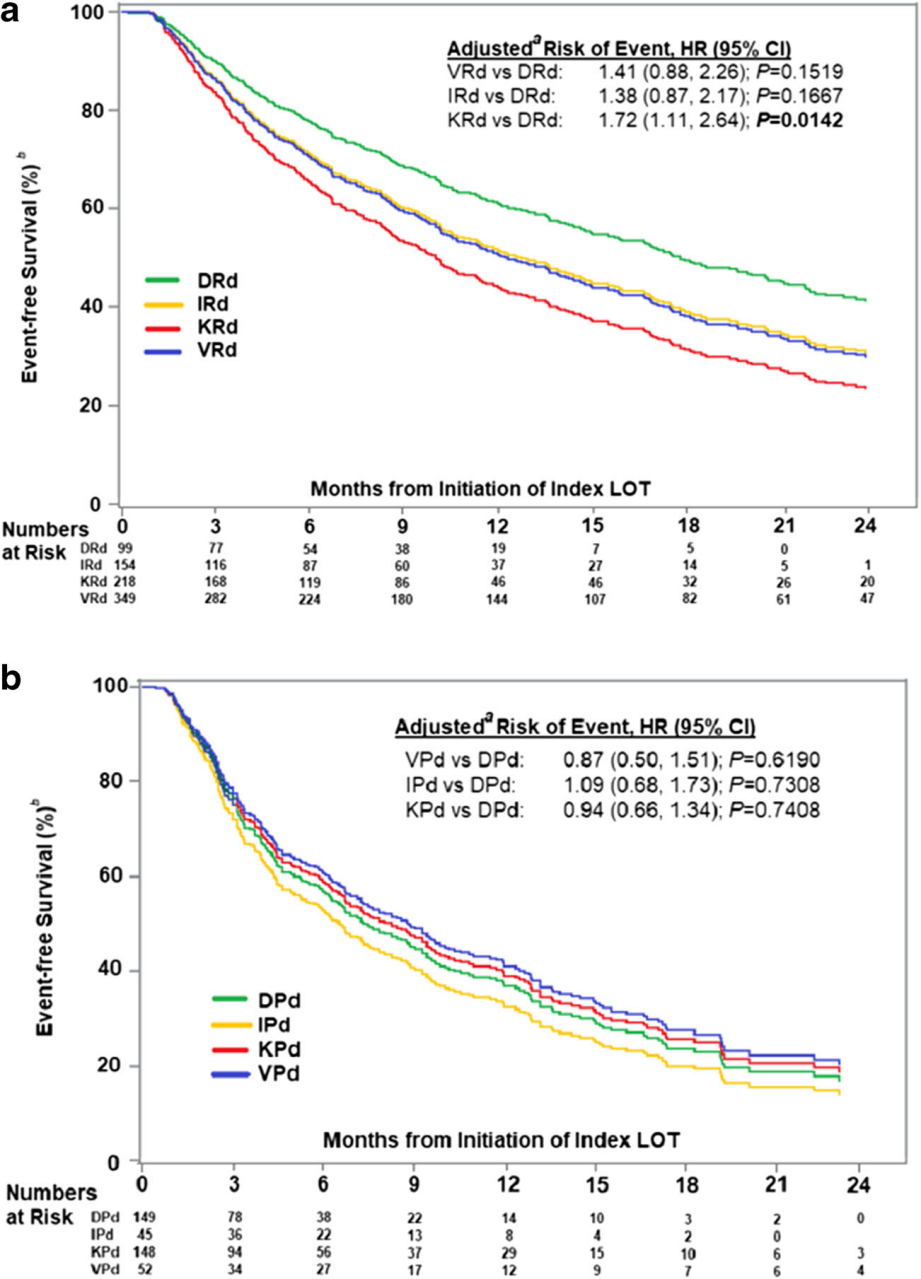
Real-world adjusted TTNT for Rd (**a**) and for Pd backbone triplets in LOT ≥ 2 (**b**), by regimen type. ^a^All survival analyses were stratified by LOT (2, 3, ≥ 4). Adjusted for age (18–64, 65–74, ≥ 75 years); CCI (0, 1, ≥ 2); EGOG PS (0–1, 2–4, unknown), presence of CRAB symptoms, presence of peripheral neuropathy, and prior SCT; cytogenetic risk (standard risk/unknown, high risk); ISS stage (I/II, III, unknown); prior treatment exposure (PI, IMID, PI+IMID, and none), prior daratumumab exposure; PI- and/or IMID-refractory status (PI-refractory, IMID-refractory, PI + IMID-refractory, and not refractory); time from diagnosis to start of index line of therapy (months); refractory to prior line of therapy (TFI of ≤ 60 days vs > 60 days); time from start of LOT1 to start of LOT2 (months); year of diagnosis (2007–2011, 2012–2015, 2016–2017); and setting of care (community, academic, unknown). ^b^An event was defined as the start of next line of therapy or death. Key: CRAB, hypercalcemia, renal insufficiency, anemia, bone disease; DRd, daratumumab, lenalidomide, dexamethasone; IMID, immunomodulator; IRd, ixazomib, lenalidomide, dexamethasone; ISS, International Staging System; KM, Kaplan-Meier; KRd, carfilzomib, lenalidomide, dexamethasone; LOT, line of therapy; PI, proteasome inhibitor; PFS, progression-free survival; Rd, lenalidomide, dexamethasone; SCT, stem cell transplant; TFI, treatment-free interval; TTNT, time to next therapy; VRd, bortezomib, lenalidomide, dexamethasone

After adjustment for baseline confounders to distinguish differences based on treatment effect, TTNT was longest among those treated with the newest Rd combination triplet, DRd, albeit the differences in risks of initiation of next line of therapy or death in patients treated with either VRd or IRd vs DRd in LOT ≥2 did not reach statistical significance (*P*>0.05; Fig. 3). However, there was a significantly higher risk of next LOT initiation or death for patients receiving KRd vs DRd (HR: 1.72; *P* = 0.0142). Adjusted median TTNT for each Rd combination triplet was as follows: IRd, 12.7 months; VRd, 12.3 months; KRd, 10.0 months; DRd, not estimable due to shorter follow-up.

Further, no regimen offered a significantly different risk of next treatment initiation or death vs D-based-triplet when combined with a Pd backbone in LOT ≥ 2, although the sample sizes in the VPd and IPd groups were small (*n* = 52 and *n* = 45, respectively). Adjusted median TTNTs were 8.8 months for VPd, 8.3 months for KPd, 6.5 months for IPd, and NE due to shorter follow-up for DPd.

Due to small sample sizes, adjusted analyses were not performed for either backbone (Rd or Pd) for patients receiving therapy in earlier LOTs (LOTs 2–3).

## Discussion

To our knowledge, this is the first large database analysis evaluating the comparative effectiveness of the PI agents available for patients with RRMM (ixazomib, carfilzomib, and bortezomib) in combination with either Rd or Pd backbones vs monoclonal antibody agent, daratumumab-based triplet therapy in a general population from a real-world dataset.

Our analysis points to several factors that may affect treatment choice. For example, a lower proportion of patients selected for either VRd or VPd compared to other agents in combination with Rd or Pd backbones had previous exposure to both a PI and an IMID or refractory status to both classes of drugs. This may be in large part due to bortezomib-based regimens being utilized in earlier lines of therapy than the other triplet combinations. Additionally, a higher proportion of patients selected for I-based therapy (both IRd and IPd) were older (≥ 75 years of age) than those selected for K-based or D-based triplet regimens in combination with Rd and Pd backbones. However, the data suggest that more patients receiving I-based triplets had asymptomatic relapse at start of therapy, indicated by a relatively greater proportion of IRd and IPd patients with no reported CRAB symptoms compared to those treated with the other agents in combination with Rd and Pd backbones.

These data highlight the important efficacy/effectiveness gap between results observed in clinical trials and those realized in the real world, as all patients selected for treatment with Rd backbone regimens experienced shorter TTNT than the PFS reported in their respective hallmark trials, with the exception of VRd, which was previously evaluated in a small single-arm, phase 2 study. Unadjusted analysis revealed that Rd triplets used in routine clinical practice have a shorter TTNT (a proxy for PFS) than the PFS reported from the hallmark clinical trials for DRd [24], IRd [5], and KRd [25] but not for VRd [26] (Fig. 2).

A number of factors can contribute to this efficacy-effectiveness gap, including differences in composition of study patients [26]. For example, all aforementioned hallmark clinical trials enrolled patients who had only received 1 to 3 lines of prior therapy (i.e., trial patients were treated in LOTs 2-4), where 47% [4] to 62% [5] of patients had only 1 prior LOT. In contrast, in the current real-world study, 27% of patients receiving DRd, 13% of patients receiving IRd, and 12% of patients receiving KRd were in LOT 5 or greater compared with 3% of those receiving VRd. Real-world exploratory analysis of patients who received one of these agents with Rd backbones in earlier LOTs 2 to 3 revealed that the unadjusted median TTNT was more consistent with these trials for IRd (*n* = 108; median TTNT 16.6 months) and was longer for VRd (*n* = 311; median TTNT: 14.1 months), but was still much shorter for KRd (*n* = 156; median TTNT 8.8 months). The median unadjusted TTNT estimate was not estimable for DRd in patients treated in LOTs 2 to 3 considering small sample size (*n* = 55) and short follow-up time (median follow-up 8.2 months).

These findings highlight the importance of patient selection. The real-world IRd patients in this study were more likely to be ≥75 years old (39.6%) than those in the TOURMALINE-MM1 phase 3 trial (13.0%) [5], and conversely, IRd was utilized in patients less likely to have CRAB symptoms at initiation of therapy than the other Rd backbone regimens. Additionally, KRd was utilized in a population in this real-world study with more renal insufficiency (42.7%; defined as a creatinine clearance [CrCl] < 40 mL/min or a serum creatinine of > 2 mg/dL) and known high-risk cytogenetics (25.7%) than in the phase 3 trial, ASPIRE (6.3%; defined as CrCl ≤ 30 mL/min; 12.1%, high-risk cytogenetics, respectively) [4]. VRd in RRMM was studied as a small phase 2 trial (*n* = 64), where 36% of the patients had relapsed after SCT, prior IMID exposure was predominantly limited to thalidomide, and patients had a median of 2 prior LOTs, unlike our population where 69% had only 1 previous LOT and only 16% had a previous SCT [26]. Finally, DRd use in routine clinical care occurred in later lines than in the phase 3 POLLUX trial, and almost a quarter of real-world DRd patients (24.2%) had received prior daratumumab (a population that was excluded in POLLUX) [6].

These differences in our real-world population of RRMM patients on a PI- or daratumumab-Rd regimen vs characteristics of the patient populations within the respective randomized, controlled trials are consistent with previously reported evaluations. A study of 788 RRMM patients receiving Rd noted that the majority of patients in the real world do not meet the eligibility criteria within the hallmark randomized trials of these agents paired with an Rd backbone; ineligibility due to patient baseline characteristics, including those noted above, ranged from 52.2% of real-world patients based on the POLLUX trial of DRd vs Rd to 72.3% based on the ASPIRE trial of KRd vs Rd [11].

After adjusting for observable confounders to isolate the treatment effect, compared to the DRd triplet regimen, neither IRd nor VRd was statistically different in terms of risk of initiating the next LOT or death, whereas patients receiving KRd experienced a significantly higher risk of next LOT initiation or death vs DRd. In the analyses of the Pd backbone-containing triplets, these real-world data suggest that no regimen in this study offered a significantly different risk of next treatment initiation or death vs DPd. Of note, the few recent RCTs comparing one triplet to another, albeit in the newly diagnosed setting, have yielded similar comparable PFS results, including ENDURANCE comparing KRd to VRd and CLARION comparing K vs V in combination with a melphalan and prednisone backbone (KMP vs VMP) [27, 28]. Both studies demonstrated no differences in PFS, although the toxicity profiles differed as expected based on the side effect profiles of K vs V. To date, in the RRMM setting, registration studies consistently compare triplets to doublets, a strategy that is increasingly difficult to justify in the future given the plethora of high-quality phase 3 studies that consistently demonstrate triplet superiority.

In tandem, these data highlight the need for further evaluation in both the RCT and real-world settings of these agents (V, K, D, and I) and their use with both IMID/dexamethasone backbones (Rd and Pd) to further elucidate the comparative efficacy and translation of efficacy results from RCTs to effectiveness in the real world to better inform individual treatment decision-making.

The limitations of this study include the possibility of residual confounding due to unobserved treatment selection biases that are inherent to any nonrandomized, observational study. Cytogenetic abnormalities, ISS stage, and ECOG PS were not available for a majority of patients. However, we attempted to minimize any bias in our multivariate analyses stemming from missing values for these prognostic factors by incorporating other covariates that are related to the kinetics of disease aggressiveness (i.e., functional risk), including time from initiation of frontline therapy to first relapse, time from diagnosis to initiation of the index LOT, and LOT number. Additionally, this study was intended to reflect real-world treatment utilization, and as such, utilized a patient LOT-based analysis; however, a sensitivity analysis looking at a patient-level evaluation has been previously conducted [29] and revealed consistent results between LOT-based and patient-level comparative effectiveness analyses. Further, refractory status to either a PI or IMID was defined using a proxy and may over- or underestimate the true proportion of refractory patients. Finally, analyses were not powered for statistical comparisons, and there were small sample sizes in some of the subgroup analyses, along with a shorter follow-up across groups for the D-based triplets relative to the other triplet groups.

In conclusion, treatment selection for use of the agents (V, K, D, and I) with an Rd backbone in routine clinical practice indicates a gap between treatment efficacy as reported in a clinical trial setting compared to effectiveness of these triplet combinations in the real world. Notably, this gap does narrow when considering real-world treatment in earlier LOTs, which is more reflective of how these regimens were studied in clinical trials for IRd and VRd but not for KRd; this was not evaluable in the DRd group. The real-world confounder-adjusted comparative effectiveness analyses of these regimens revealed that PI-Rd and PI-Pd triplets were comparable to DRd and DPd, respectively, in terms of risk of next LOT initiation or death, except for KRd, which resulted in a higher risk of start of next LOT or death compared to DRd. Considering the ever-evolving treatment landscape of MM in routine clinical practice, the need for further research on real-world comparative effectiveness of triplet therapies for management of MM is necessary to both confirm prior findings and report findings based on the most current routine patterns of care.

## Supplementary information

Supplementary Figure 1. Study Design Schema. Key: LOT – line of therapy. (DOCX 45 kb).

ESM 1 (DOCX 17 kb).

## Data Availability

Data for this study was made available through a third-party license from Optum, a commercial data provider in the US; our analysis and results are reported in the main text and accompanying appendix. Further release of the dataset is not possible due to a data use agreement.
