# Supplementary material for: Real-world comparative effectiveness of triplets containing bortezomib (B), carfilzomib (C), daratumumab (D), or ixazomib (I) in relapsed/refractory multiple myeloma (RRMM) in the US
Source: Ann Hematol. 2021 May 10;100(9):2325–37. doi: 10.1007/s00277-021-04534-8 (PMC8357697; doi:10.1007/s00277-021-04534-8)
Supplement: Supplementary file 1 — Study Design Schema. Key: LOT – line of therapy. (DOCX 45 kb). [file 277_2021_4534_MOESM1_ESM.docx]

**Suppl Fig 1.**

**Index Treatment Start Date LOT ≥2**

6-month baseline period

Index treatment exposure: Jan. 1, 2014 to Mar 31, 2018

**Follow-up period**

March 31, 2018

**Study Period: Jan 1, 2007 to March 31, 2018**

**Start enrollment**

End enrollment

Jan 1, 2007

July 1, 2007
