# Supplementary material for: Real-world comparative effectiveness of triplets containing bortezomib (B), carfilzomib (C), daratumumab (D), or ixazomib (I) in relapsed/refractory multiple myeloma (RRMM) in the US
Source: Ann Hematol. 2021 May 10;100(9):2325–37. doi: 10.1007/s00277-021-04534-8 (PMC8357697; doi:10.1007/s00277-021-04534-8)
Supplement: Supplementary file 2 — (DOCX 17 kb). [file 277_2021_4534_MOESM2_ESM.docx]

**SUPPLEMENTARY APPENDIX**

***Identification of LOTs***

LOT was determined per the treatment algorithm outlined below, which utilizes a comprehensive view of treatment patterns per the National Comprehensive Cancer Network^®^ (NCCN) Guidelines for Multiple Myeloma [9]. It should be noted that updated versions (i.e., as of version 3.2017) of the guidelines for MM removed all melphalan-containing regimens, thalidomide/‌dexamethasone, liposomal doxorubicin/vincristine/dexamethasone, and vincristine/‌doxorubicin/dexamethasone for non-SCT candidates and thalidomide/‌‌‌dexamethasone, single-agent dexamethasone, and vincristine/‌doxorubicin/‌dexamethasone for SCT candidates. However, due to the time frame of these data, these regimens were included. The treatment algorithm is summarized below.

Patients with an SCT (within 300 days of first-line regimen):

- Induction therapy received prior to SCT was part of the first-line regimen.
- Continuation of the same or a subset of the induction regimen was continuation of first line, unless the interval between re-treatment and most recent prior regimen was at least 6 months, in which case re-treatment constituted second-line treatment.
- Start of single-agent lenalidomide or bortezomib within 12 months after SCT was part of first-line maintenance therapy.
- The switch/addition of a new drug (not including steroids) after a 60-day gap from SCT was second-line treatment.

Patients with no SCT (within 300 days of first-line regimen):

- Drugs initiated within 90 days of the first date for an MM-specific anticancer agent after first MM diagnosis date constituted the initial frontline regimen.
- Continuation of the same regimen or subset thereof was part of first-line treatment unless the interval between re-treatment and the most recent prior regimen was at least 6 months, in which case re-treatment constituted second-line treatment.
- Start of single-agent lenalidomide or bortezomib within 6 months of the end of initial therapy was part of first-line maintenance therapy.
- Switch/addition of a new drug (not including steroids) compared to initial therapy was second-line treatment.

For all patients, subsequent lines of therapy (third and beyond) occurred if:

- There was a switch/addition of a new MM-specific anticancer drug (not including steroids) compared to the regimen in prior line, or
- Re-treatment with the same regimen/subset where a gap between the end of the prior regimen and start of re-treatment was at least 6 months.
- Single-agent dexamethasone (but not prednisone) constituted a regimen if dexamethasone alone was >90 days in duration.
